# Safety and Long-Term Outcomes of Rotablation in Patients with Reduced (<50%) Left Ventricular Ejection Fraction (rEF) (The Rota-REF Study)

**DOI:** 10.3390/jcm12175640

**Published:** 2023-08-30

**Authors:** Mohamed Ayoub, Péter Tajti, Ibrahim Akin, Michael Behnes, Tobias Schupp, Jan Forner, Hazem Omran, Dirk Westermann, Volker Rudolph, Kambis Mashayekhi

**Affiliations:** 1 Clinic for General and Interventional Cardiology/Angiology, Heart and Diabetes Center NRW, Ruhr University Bochum, 32545 Bad Oeynhausen, Germany; 2 Gottsegen György National Cardiovascular Center, 1096 Budapest, Hungary; 3 Department of Cardiology, Angiology, Haemostaseology and Medical Intensive Care, University Medical Centre Mannheim, Medical Faculty Mannheim, Heidelberg University, 68167 Mannheim, Germany; 4 Department of Cardiology and Angiology II, University Heart Center Freiburg, 79189 Bad Krozingen, Germany; 5 Department of Internal Medicine and Cardiology, Mediclin Heart Centre Lahr, 77933 Lahr, Germany

**Keywords:** percutaneous coronary intervention, rotational atherectomy, coronary artery disease, reduced left ventricular ejection fraction

## Abstract

Clinical outcomes in patients with reduced left ventricular systolic function undergoing rotational atherectomy (RA) for percutaneous coronary intervention (PCI) remain understudied. Our study sought to evaluate the impact of RA-PCI in patients with LV systolic dysfunction on long-term outcomes. Between 2015 and 2019, 4941 patients with reduced LV function (rEF) undergoing PCI (with or without RA) were included in the hospital database. The primary endpoint was in-hospital major adverse cardiovascular and cerebral events (MACCE). The secondary endpoint was 3-year MACCE. In-hospital MACCE rates were significantly higher in RA-PCI compared to standard PCI without RA (PCI) (7.6% vs. 3.9%, *p* = 0.0009). However, 3-years MACCE rates were similar in RA-PCI and PCI (26.40% vs. 26.6%, *p* = 0.948). In conclusion, RA-PCI in patients with rEF is feasible, safe, and shows similar long-term results to PCI.

## 1. Introduction

The number of patients with calcified coronary lesions treated using percutaneous coronary intervention (PCI) with rotational atherectomy (RA) has been increasing over the last decade [1]. The indication for RA varies from 11.60% to 19.6% of patients treated with moderately/severely calcified coronary lesions [1,2]. Age, diabetes mellitus, and advanced renal disease are well-known predictors of coronary calcification [3,4]. High-speed RA can prepare calcified coronary lesions effectively due to the removal of obstructive calcified atheroma by differential cutting [5]. The PREPARE-CALC study has shown the feasibility of RA with a high procedural success rate in nearly all patients with significantly calcified coronary lesions [6].

PCI of complex coronary lesions has a higher risk of stent under-expansion, restenosis, target lesion revascularization, myocardial infarction, and death [7]. Similarly, reduced left ventricular ejection fraction (rEF) is associated with a higher rate of major adverse cardiovascular and cerebral events (MACCE) and mortality after PCI [8]. Multiple retrospective analyses showed a higher risk for mortality in patients with rEF who underwent PCI or PCI for chronic total occlusion (CTO), compared to patients with preserved EF [9,10,11,12,13]. 

This study aims to investigate the safety and long-term outcomes of RA-PCI in patients with rEF.

## 2. Materials and Methods

All patients undergoing PCI at a tertiary heart center were consecutively enrolled in a dedicated PCI Registry. Data collection included all patients with preserved and reduced left ventricular ejection fraction (LVEF). Reduced LVEF was defined as LVEF < 50%. A total of 14.592 patients who underwent PCI between January 2015 and December 2019 were included in the hospital database. Out of these, 4941 patients had rEF and were included in our analysis. Indication for coronary intervention was based on symptoms (angina pectoris) or reproducible ischemia on functional stress testing or fractional flow reserve according to the European Guidelines for Myocardial Revascularization [14]. Noninvasive measurement of LV function by echocardiography was performed in most patients. Patients were followed up prospectively by telephone interview at 30 days, 1 year, and 3 years after PCI. In addition, follow-up data were obtained from hospital re-admission records, contact with the referring physician, or outpatient visits. Written informed consent for PCI and participation in the registry was obtained from each patient. Data collection was performed as per the Declaration of Helsinki and approved by the institutional review board (Ethical approval number: EK 21-1100). All angiographic results were analyzed by two independent interventional cardiologists reviewing coronary angiograms and procedure-related data retrospectively from source data and related documentation.

Lesion complexity was classified according to the American College of Cardiology/American Heart Association Lesion Classification (ACC/AHA) as type A, B1, B2, and C lesions, described by Ryan et al. [15]. Severe calcification of the target lesion was defined by cine angiography (i.e., radiopacities noted without cardiac motion before contrast injection generally compromising both sides of the arterial lumen) [16]. The decision to perform RA was at the operators’ discretion and RA was performed initially (upfront RA) due to heavy calcification in the target lesion or as a bailout in uncrossable calcified lesions or in the case of previously failed PCI attempts due to balloon undilatable lesions. All RA-PCIs were performed by high-volume PCI operators (i.e., defined by a minimum of *n* = 50 RA-PCIs performed each year) using the rotablation atherectomy system (Boston Scientific Corp., Natick, MA). Vascular access, burr size, and ablation speed were left to the operators’ discretion. Postinterventional 12-lead electrocardiogram was documented 24 h after PCI and cardiac biomarkers (CK, CK-MB, and Troponin) were measured after 8, 16, and 24 h. In addition, patients were clinically monitored during the entire hospital stay. 

LVEF was assessed by calculating the biplane Simpson method in 2-dimensional transthoracic echocardiography according to current recommendations of the American Society of Echocardiography and the European Association of Echocardiography [17]. 

The primary endpoint was in-hospital MACCE which included any of the following adverse events before hospital discharge: all-cause death, myocardial infarction, recurrent symptoms requiring urgent target vessel revascularization (TVR) or target lesion revascularization (TLR) with PCI or surgery, and stroke. Secondary endpoints included 1-year and 3-year MACCE. Additional secondary procedural endpoints regarding procedural and fluoroscopy time, radiation, and contrast dose, as well as major complications, like vascular access site complications and pericardiocentesis, were also reported. Myocardial infarction (MI) was defined using the 4th universal definition (type 4a) described by Thygesen et al. [18]. Procedural success was defined as technical success without in-hospital MACCE. Technical success was defined as successful revascularization of occlusive and non-occlusive coronary lesions with the achievement of <30% residual diameter stenosis within the treated segment and restoration or maintenance of TIMI grade 3 antegrade flow. 

### Statistical Methods

Continuous data were presented as mean ± standard deviation or median and interquartile range (IQR) unless otherwise specified, and were compared using the Student *t*-test. The Wilcoxon rank-sum test and the Kruskal–Wallis tests were applied for non-parametric continuous variables, as appropriate. Categorical variables were expressed as percentages and were compared using Pearson’s chi-squared test or Fisher’s exact test. Multivariable analyses were calculated at baseline for the prediction of all-cause MACCE by using Cox regression with backward elimination. Crude and adjusted hazard ratios with 95% confidence intervals (95% CI) were calculated after the selection of the confounding variables based on univariable association with the given endpoints in the present study (*p* < 0.05). Cumulative event rates were calculated according to the Kaplan–Meier method, and comparisons were performed with the log-rank test. A *p*-value of <0.05 was considered statistically significant, and all *p*-values were 2-sided. All statistical analyses were performed with JMP 13.0 (SAS, Cary, NC, USA).

## 3. Results

### 3.1. Baseline Angiographic and Procedural Characteristics 

Out of the 4.941 patients with rEF treated with PCI, 197 (3.99%) underwent RA-PCI. Patients with RA-PCI were older and had higher rates of diabetes, renal dysfunction, hypertension, prior CABG, CTO vessel, and a history of myocardial infarction compared to patients with standard PCI. On the other hand, patients with RA-PCI had a lower rate of ACS presentation, a lower rate of smoking, and lower LDL levels than the standard PCI group. Baseline characteristics are shown in (Table 1). 

Overall, lesions in the RA-PCI group were markedly more complex. In comparison to the PCI group, the RA-PCI group contained more left main PCIs (*p* < 0.0001), and more type c lesions (*p* < 0.001). Coronary lesions were also longer (*p* < 0.0001) and more severely calcified (*p* < 0.0001) (Table 2). Therefore, procedures performed with RA required a significantly higher mean number of implanted stents per patient (*p* < 0.0001), longer overall stent length (*p* < 0.0001), and bigger sheets (7Fr and 8Fr) (*p* < 0.0001). Moreover, femoral access was used more often in the RA-PCI group (*p* < 0.0001) (Table 3). 

### 3.2. Primary and Secondary Outcomes

**Figure 1:**
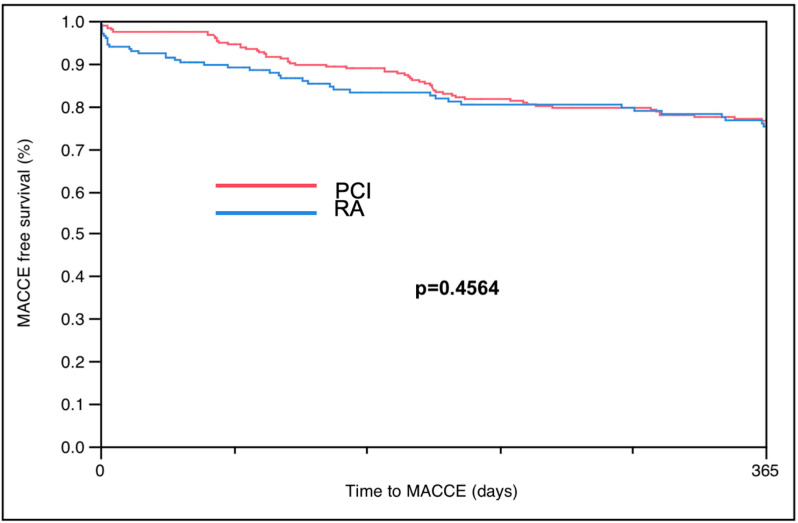
Kaplan–Meier curve for patients with LVEF< 50%.

**Figure 2:**
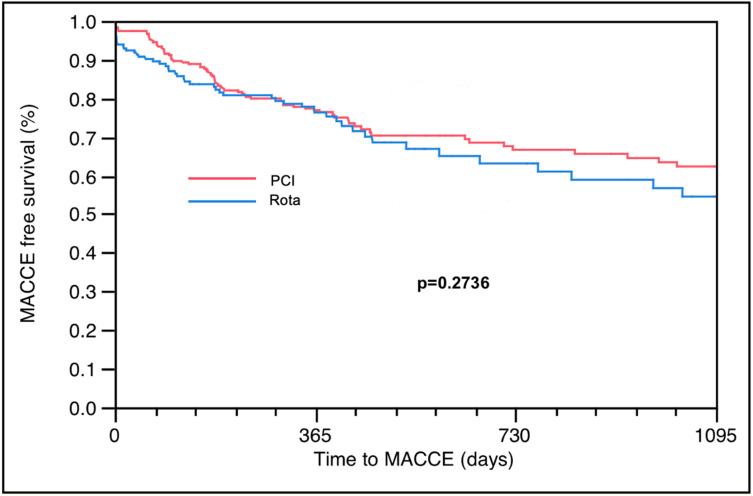
Three-year MACCE-free survival in patients undergoing rotational atherectomy with LVEF< 50%.

The overall procedural success rate was 93.10%, with no significant difference between PCI and RA-PCI (93.1% vs. 92.4%, *p* = 0.687) (Table 4). In-hospital MACCE rates were significantly higher in the RA-PCI group compared to the PCI group (7.6% vs. 3.9%, *p* = 0.0009). There was only a trend for increased in-hospital mortality with RA-PCI compared to PCI (4.6% vs. 2.4%, *p* = 0.062) (Table 4). However, 1-year MACCE in the Kaplan–Meier curve (Figure 1) and 3-year MACCE rates did not differ in the RA-PCI compared to the PCI group (26.4% vs. 26.6%, *p* = 0.9) (Figure 2) (Table 4).

Procedural complications were generally rare. Coronary perforation requiring pericardiocentesis was higher in RA-PCI compared to PCI (1.5% vs. 0.23%, *p* = 0.0008) (Table 4). In addition, RA-PCI was associated with significantly longer procedural time (*p* < 0.0001), longer fluoroscopy time (*p* < 0.0001), higher contrast volume (*p* < 0.0001) and higher radiation dose (*p* < 0.0001). 

After multivariable adjustment for baseline differences, impaired renal function (*p* < 0.0001), acute coronary syndrome presentation (*p* < 0.0001), higher LDL level (*p* = 0.0072), and severe calcification (*p* < 0.0001) were independent predictors of mortality in the PCI (Table 5). 

On the other hand, only ACS presentation (*p* = 0.0139) and prior CABG (*p* = 0.0438) were independent predictors of mortality in the RA-PCI group (Table 6). 

## 4. Discussion

The major findings of our study were that patients who underwent RA-PCI were older, had more co-morbidities, especially chronic renal insufficiency, and also had advanced coronary artery disease as represented by higher rates of previous CABG and more complex lesions. This translated into increased procedural and fluoroscopy times and increased contrast medium use along with an increase in vascular complication rates and in-hospital MACCE. Nevertheless, procedural success rates of RA-PCI were very high. Moreover, despite the higher in-hospital MACCE rate in the RA-PCI group, in-hospital mortality as well as the 3-year MACCE rate did not significantly differ between RA-PCI and PCI. Our findings underline the feasibility and safety of RA-PCI in patients with reduced LVEF.

There are many possible explanations why a reduced LVEF adversely affects outcomes in patients who have undergone RA-PCI. Patients with reduced LVEF are usually multimorbid and, as such, at a higher risk of complications [10]. Several studies have shown a significant correlation between reduced LVEF and mortality in patients who underwent PCI [11,12,13], which is also noted in our analysis. In addition, coronary microvascular obstruction resulting from distal embolization of the released debris during the RA procedure might further worsen the LVEF [19]. And thermal injury as well as thrombocyte activation might propagate microvascular dysfunction and consequently result in more periprocedural myocardial injury and infarction [20]. Despite the higher in-hospital MACCE rate in patients with RA-PCI, this was not associated with increased mortality, which is reassuring. A possible explanation for this is the trade-off between the increased procedural complexity with potentially higher complication rates on the one hand, and the better lesion preparation through debulking before stenting to achieve better stent expansion, which leads to better long-term outcomes on the other [21,22]. Furthermore, multivariate cox regression analysis showed that ACS presentation, prior CABG, and renal dysfunction were the only independent predictors of mortality in patients with reduced LVEF. Interestingly, moderate to severe calcifications were associated with increased long-term mortality only in patients who underwent PCI without RA, but not in patients who underwent RA-PCI. This underlines the efficacy of RA-PCI in severely calcified lesions and ensures its safety.

## 5. Limitations

Our study has several limitations. First, it is based on a retrospective non-randomized single-center registry. Furthermore, the indication for RA-PCI and the selected device was left to the operator’s discretion and may have varied depending upon their technical expertise and experience, which makes selection bias possible. That is why we performed a regression analysis adjusting for baseline covariates trying to minimize those differences. Also, we relied on angiographic evidence of calcification without routine use of intravascular imaging, which should be the standard of care for calcium quantification and optimal modification. More studies are needed to confirm our results.

## 6. Conclusion

In conclusion, our study demonstrates the utility, efficacy, and safety of RA-PCI in patients with complex coronary artery disease despite reduced LVEF. 

## Figures and Tables

**Table 1:** Baseline characteristics with rEF undergoing PCI and RA-PCI.

		LVEF < 50%		
Patient Characteristics	Overall %	PCI	Rota	*p* Value
	(*n* = 4941)	(*n* = 4744)	(*n* = 197)	
Age (years)	70.61 ± 10.99	70.5 ± 11.0	73.7 ± 9.3	0.0001 *
Men	81.2% (4016)	81.2% (3854)	82.2% (162)	0.726
BMI (kg/m^2^)	27.68 ± 4.58	27.7 ± 4.6	27.9 ± 4.0	0.415
eGFR, ml/min/1.73 m^2^	64.67 ± 23.20	64.92	58.68	0.0002 *
Heart Failure	14.8% (659)	14.86% (636)	13.61% (23)	0.653
NYHA Classification				0.340
• NYHA I	13.45% (541)	13.62% (524)	9.83% (17)	
• NYHA II	37% (1488)	37% (1424)	37% (64)	
• NYHA III	38.82% (1561)	38.59% (1485)	43.93% (76)	
• NYHA IV	10.72% (431)	10.78% (415)	9.25% (16)	
LVEF				0.825
41–49%	55.45% (2740)	55.52% (2634)	53.81% (106)	
30–40%	27.52% (1360)	27.45 (1302)	29.44 (58)	
<30%	17.02% (841)	17.03% (808)	16.75% (33)	
CAD presentation				0.12
ACS	32.99% (1630)	33.20% (1575)	27.92% (55)	
No ACS	67% (3311)	66.8% (3169)	72.08% (142)	
CTO vessel	22.1% (1090)	21.33% (1010)	40.61% (80)	0.008 *
Diabetes mellitus	35.9% (1623)	35.3% (1533)	50.0% (90)	0.0001 *
Dyslipidemia	87.1% (3945)	86.9% (3779)	91.7% (166)	0.057
Hypertension	87.6% (4138)	87.4% (3962)	92.6% (190)	0.033 *
Current Smoker	17.7% (809)	18.0% (794)	8.4% (15)	0.0009 *
Family History of CAD	36.8% (1481)	37.1% (1441)	27.8% (40)	0.022 *
Prior Myocardial Infarction	43.3% (1894)	43.0% (1807)	51.8% (87)	0.024 *
Prior CABG	20.1% (910)	19.1% (829)	45.5% (81)	0.0001 *
LDL Max., mg/dL	106.14 ± 41.8	106.8 ± 42.0	90.5 ± 31.1	0.0001 *
Positive stress test	78.8% (1455)	78.6% (1414)	87.2% (41)	0.150

Values are given as percentages of patients and numbers or as mean and standard deviation. CABG = coronary artery bypass grafting, CAD = coronary artery disease, CTO = chronic total occlusion, eGFR = estimated glomerular filtration rate, LDL = low-density lipoprotein, LVEF = left ventricular ejection fraction, PCI = percutaneous coronary intervention, BMI = body mass index, * statistically significant with *p* < 0.05.

**Table 2:** Angiographic characteristics in patients with rEF stratified for PCI and RA-PCI.

		LVEF < 50%		
Lesion Characteristics	Overall %	PCI	Rota	*p* Value
	(*n* = 4941)	(*n* = 4744)	(*n* = 197)	
Target vessel				0.0001 *
Left main	6.7% (330)	6.2% (295)	17.8% (35)	
Right coronary artery	34.9% (1723)	34.7% (1645)	39.6% (78)	
Left circumflex artery	19.6% (970)	19.7% (937)	16.7% (33)	
Left anterior descending artery	36.1% (1786)	36.6% (1737)	24.9% (49)	
SVG	2.6% (128)	2.6% (126)	1.0% (2)	
Lesion length, mm				
<10 mm	11.4% (548)	11.7% (541)	3.6% (7)	0.0001 *
10–20 mm	40.6% (1959)	41.1% (1901)	29.9% (58)	
>20 mm	47.59% (2315)	47.2% (2186)	66.5% (129)	
AHA/ACC classification				0.0001 *
A/B1	23.2% (1147)	24.1% (1140)	3.6% (7)	
B2	28.1% (1389)	28.7% (1359)	15.2% (30)	
C	48.6% (2397)	47.2% (2237)	81.2% (160)	
Calcification				0.0001 *
None	7.8% (386)	8.2% (386)	0,0% (0)	
Mild	42.9% (2113)	44.6% (2108)	2.5% (5)	
Moderate	26.8% (1319)	27.6% (1306)	6.6% (13)	
Severe	22.5% (1107)	19.6% (928)	90.9% (179)	
Eccentric calcification	70.9% (3262)	71.4% (3152)	57.9% (110)	0.0001 *
Tortuosity	16.0% (785)	15.6% (734)	25.9% (51)	0.0001 *
Relevant side branch	29.4% (1432)	29.5% (1382)	26% (50)	0.297
Intra-lesion Angulation				0.0001 *
none	10.5% (514)	10.2% (483)	15.7% (31)	
<45%	49.4% (2428)	50% (2359)	35% (69)	
45–90%	36% (1768)	35.8% (1689)	40.1% (79)	
>90%	4.1% (203)	3.9% (185)	9.1% (18)	

Values are given as percentages of patients and numbers. CTO = chronic total occlusion, LAD = left anterior descending coronary artery, LCX = left circumflex coronary artery, LM = left main coronary artery, RCA = right coronary artery, SVG = single vein graft, * statistically significant with *p* < 0.05.

**Table 3:** Technical characteristics in patients with rEF stratified for PCI and RA- PCI.

		LVEF < 50%		
Overall Procedural Results	Overall	PCI	Rota	*p* Value
	(*n* = 4941)	(*n* = 4744)	(*n* = 197)	
Balloon diameter pre-dilatation, mm	2.6 ± 0.79	2.6 ± 0.9	3.3 ± 0.9	0.0001 *
Balloon diameter post-dilatation, mm	3.8 ± 0.7	3.7 ± 1.1	3.93 ± 1.28	0.0001 *
Inflation pressure pre-dilatation, atm	16.5 ± 5.1	16.4 ± 6.3	21 ± 6.3	0.0001 *
Inflation pressure post-dilatation, atm	19.8 ± 5.1	19.71 ± 5.1	21.9 ± 5.8	0.0001 *
Diameter stenosis pre PCI, %	86 ± 14	86.7% ± 14.1	87.5% ± 14.8	0.108
Diameter stenosis post PCI, %	5 ± 19	5.1% ± 20	3.7% ± 15.6	0.980
Number of stents implanted	1.26 ± 0.88	1.2 ± 0.9	1.9 ± 1.2	0.0001 *
Stent diameter in mm	3.2 ± 1.44	3.2 ± 1.9	3.6 ± 1.4	0.0001 *
Overall stent length, mm	35.4 ± 26.1	32 ± 23.9	53.1 ± 31.6	0.0001 *
Burr Size used				0.0001 *
• 1.25 mm			23.2% (39)	
• 1.50 mm			41.7% (70)	
• 1.75 mm			30.4% (51)	
• 2.00 mm			4.8% (8)	
Access site				0.0001 *
Single Radial access	56.55% (2675)	57.61% (2620)	30.22% (55)	
Any femoral access	42.79% (2024)	41.75% (1899)	68.7% (125)	
Guiding catheter size				0.0001 *
6Fr	83.79% (3965)	86.07 (3916)	26.92% (49)	
7Fr	14.14% (669)	12.13% (552)	64.29% (117)	
8Fr	1.18% (56)	0.95% (43)	7.14% (13)	

Values are given as percentages of patients and numbers or as median and interquartile range. PCI = percutaneous coronary intervention, * statistically significant with *p* < 0.05.

**Table 4:** Study endpoints, procedural results, and major complications in patients with rEF stratified for PCI and RA-PCI.

		LVEF < 50%		
	Total Number (*n* = 4941)	PCI (*n* = 4744)	Rota (*n* = 197)	*p*-Value
Primary endpoint % (*n*)				
Procedural Success	93.10% (4600)	93.1% (4418)	92.4% (182)	0.687
Technical success	96.1% (4748)	96% (4555)	98% (193)	0.482
In-hospital MACCE	4% (200)	3.9% (185)	7.6% (15)	0.009 *
Mortality	2.5% (125)	2.4% (116)	4.6% (9)	0.062
MI SCAI	16.07% (785)	15.38% (751)	17.26% (34)	0.548
TVR	3.4% (167)	3.3% (156)	5.6% (11)	0.080
Stroke	0.26% (13)	0.25% (12)	0.5% (1)	0.411
TLR	3.2% (160)	3.1% (149)	5.6% (11)	0.057
1-year MACCE	18.84% (931)	18.74% (889)	21.32% (42)	0.364
3-year MACCE	26.6% (1314)	26.6% (1262)	26.4% (52)	0.949
No flow (TIMI 0)	0.5% (26)	0.5% (24)	1% (2)	0.27
Slow flow (TIMI 1 or 2)	4.9% (244)	5% (239)	2.5% (5)	0.11
Secondary procedural endpoints % (*n*)				
Procedural time (min)	44 (26–75)	42 (26–72)	100 (70–136.5)	0.0001 *
Fluoroscopy time (min)	16 (10–29)	16 (9.5–28)	39 (27–62.5)	0.0001 *
Contrast volume used (mL)	197.5 (150–270)	190 (150–260)	250 (182.5–345)	0.0001 *
Dose Area Product (cGy · cm^2^)	5991 (3635–9863)	5884.5 (3492–9669)	9137 (5516–15,921)	0.0001 *
Major Complications % (*n*)				
Pericardiocentesis	0.28% (14)	0.23% (11)	1.5% (3)	0.0008 *
Vascular access complication	1.74% (63)	1.69% (60)	3.75% (3)	0.1608

Values are given as percentages of patients and numbers or as median and interquartile range. MI = myocardial infarction, TIMI = thrombolysis in myocardial infarction, TVR = target vessel revascularization, TLR = target lesion revascularization, MACCE = major adverse cardiac and cerebrovascular events, * statistically significant with *p* < 0.05.

**Table 5:** Cox regression analyses predicting long-term all-cause mortality (at 3 years) in patients presenting with rEF undergoing PCI.

Variable	Crude HR			Adjusted HR		
	HR	95% CI	*p* Value	HR	95% CI	*p* Value
Age	1.02	1.02–1.01	<0.0001	-	-	-
Men	1.07	1.22–0.93	0.321	-	-	-
eGFR per unit mL/min/1.73 m^2^	0.98	0.99–0.98	<0.0001	0.98	0.99–0.98	0.0001 *
ACS	1.62	1.81–1.45	<0.0001	1.66	1.86–1.49	0.0001 *
Diabetes mellitus	1.54	1.72–1.37	<0.0001	-	-	-
Hypertension	1.34	1.62–1.12	0.0009	-	-	-
Current smoker	0.90	1.05–0.78	0.202	-	-	-
Prior CABG	1.40	1.59–1.23	<0.0001	-	-	-
LDL Max., mg/dL	0.99	1.00–0.99	0.0004	0.98	0.99–0.98	0.0072
Positive stress test	1.31	1.64–1.06	0.0110 *	-	-	-
Target vessel				-	-	-
Lesion length	1.05	1.17–0.95	0.321	-	-	-
AHA/ACC classification	1.19	1.35–1.05	0.006 *	-	-	-
Calcification	1.40	1.55–1.25	<0.0001	1.35	1.50–1.21	0.0001 *
Eccentric calcification	1.22	1.39–1.06	0.0034 *	-	-	-
Intra-lesion angulation	0.95	1.06–0.85	0.361	-	-	-

CABG = coronary artery bypass grafting, CAD = coronary artery disease, LVEF = left ventricular ejection fraction, CI = confidence interval, HR = hazard ratio, * statistically significant with *p* < 0.05.

**Table 6:** Cox regression analyses predicting long-term all-cause mortality (at 3 years) in patients presenting with rEF undergoing RA-PCI.

Variable	Univariable			Multivariable Model		
	HR	95% CI	*p* Value	HR	95% CI	*p* Value
Age	0.99	1.02–0.97	0.767	-	-	-
Men	1.71	3.13–0.88	0.106	-	-	-
eGFR per unit mL/min/1.73 m^2^	0.99	1.00–0.98	0.699	-	-	-
ACS	2.09	3.56–1.20	0.0088 *	2.11	3.77–1.17	0.0139
Diabetes mellitus	1.04	1.85–0.58	0.900	-	-	-
Hypertension	1.22	4.12–0.49	0.691	-	-	-
Current smoker	1.02	2.53–0.30	0.967	-	-	-
Prior CABG	1.72	3.11–0.97	0.063	1.81	3.27–1.01	0.0438
LDL Max., mg/dL	0.60	2.35–0.14	0.474	-	-	-
Positive stress test	2.17	39.53–0.42	0.407	-	-	-
Target vessel				-	-	-
Lesion length	0.97	1.75–0.55	0.922	-	-	-
AHA/ACC classification	1.55	27.52–0.33	0.639	-	-	-
Calcification	1.62	28.68–0.35	0.605	-	-	-
Eccentric calcification	0.58	1.02–0.33	0.058	-	-	-
Intra-lesion angulation	1.00	1.72–0.58	0.988	-	-	-

CABG = coronary artery bypass grafting, CAD = coronary artery disease, LVEF = left ventricular ejection fraction, CI = confidence interval, HR = hazard ratio, * statistically significant with *p* < 0.05.

## Data Availability

The datasets used and/or analyzed in the current study are available from the corresponding author upon reasonable request.
